# RNA-Based Assessment of Diversity and Composition of Active Archaeal Communities in the German Bight

**DOI:** 10.1155/2012/695826

**Published:** 2012-11-12

**Authors:** Bernd Wemheuer, Franziska Wemheuer, Rolf Daniel

**Affiliations:** ^1^Department of Genomic and Applied Microbiology and Göttingen Genomics Laboratory, Institute of Microbiology and Genetics, Georg-August University of Göttingen, Grisebachstraße 8, 37077 Göttingen, Germany; ^2^Section of Agricultural Entomology, Department for Crop Sciences, Georg-August University of Göttingen, Grisebachstraße 6, 37077 Göttingen, Germany

## Abstract

*Archaea* play an important role in various biogeochemical cycles. They are known extremophiles inhabiting environments such as thermal springs or hydrothermal vents. Recent studies have revealed a significant abundance of *Archaea* in moderate environments, for example, temperate sea water. Nevertheless, the composition and ecosystem function of these marine archaeal communities is largely unknown. To assess diversity and composition of active archaeal communities in the German Bight, seven marine water samples were taken and studied by RNA-based analysis of ribosomal 16S rRNA. For this purpose, total RNA was extracted from the samples and converted to cDNA. Archaeal community structures were investigated by pyrosequencing-based analysis of 16S rRNA amplicons generated from cDNA. To our knowledge, this is the first study combining next-generation sequencing and metatranscriptomics to study archaeal communities in marine habitats. The pyrosequencing-derived dataset comprised 62,045 archaeal 16S rRNA sequences. We identified *Halobacteria* as the predominant archaeal group across all samples with increased abundance in algal blooms. *Thermoplasmatales* (*Euryarchaeota*) and the Marine Group I (*Thaumarchaeota*) were identified in minor abundances. It is indicated that archaeal community patterns were influenced by environmental conditions.

## 1. Introduction

It has been calculated that one mL of oceanic sea water contains up to 10^6^ different microorganisms [1]. These archaea, bacteria, protists, and unicellular fungi contribute 98% to the primary biomass production and are involved in almost all biogeochemical cycles [2]. It has been estimated that the global ocean harbors approximately 1.3 × 10^28^ archaeal cells and 1.3 × 10^28^ bacterial cells, which together constitute 63% to 90% of the entire marine picoplankton [3]. In addition, high numbers of *Archaea* have been found in marine sediments [4].

 In contrast to their relatives living in extreme environments, little is known on marine *Archaea. *This is partly due to the unavailability of pure cultures. Marine* Archaea* might be involved in the oceanic nitrogen cycle as some marine *Crenarchaeota* are capable of nitrification [5]. However, our knowledge of the archaeal role in oceanic ecology is rudimentary and their influence on global biogeochemical cycles is largely unexplored [6].

 Culture-independent approaches have greatly advanced our knowledge of the diversity and ecology of marine microbial communities [7–9]. Next-generation sequencing (NGS) contributed to this advancement. For example, many different ecosystems such as soil [10, 11] or sea water [12] have been studied by DNA-based high throughput sequencing of 16S rRNA gene fragments and analysis of the obtained sequences. The main drawback of DNA-based metagenomic approaches is the inability to distinguish between active and inactive community members.

 Active members and functions of microbial communities are accessible by employing RNA-based metatranscriptomic approaches. For example, Urich et al. [13] analyzed the composition and metabolic potential of active soil microbial communities by sequencing of reverse transcribed total RNA. Other studies analyzed gene expression in ocean surface waters [8] or in a deep-sea hydrothermal plume [14]. However, mainly bacterial communities and their capabilities were analyzed in these studies.

 In this paper, we investigated the composition of active archaeal communities in surface water derived from the southeastern part of the North Sea, the German Bight. The northwest of the German Bight is separated from the remaining North Sea by the Doggerbank, a large sandbank. Large coastal parts of the bight are shallow with water depths of approximately 2 to 12 meters. In our investigation, we collected seven water samples at different locations and depths in these shallow offshore areas.

 The aim of our study was to assess the active archaeal community structures in the southern North Sea employing next-generation sequencing of 16S rRNA amplicons generated by reverse transcription polymerase chain reaction (RT-PCR). To our knowledge, this is the first study using this combined approach to study marine archaeal communities. 

## 2. Material and Methods

### 2.1. Sampling and Sample Preparation

Seven marine water samples were taken for archaeal community analysis. Approximately 50 liters of sea water per sampling site were collected on board of the research vessel Heincke in May 2010 employing a conductivity, temperature, and depth (CTD) profiler. All sites were located in the German Bight. Sea water samples were prefiltered through a 10 *μ*m-mesh-size nylon net and a filter sandwich consisting of a precombusted (4 h at 450°C) 47 mm-diameter glass fiber filter (Whatman GF/D; Whatman, Maidstone, UK) and a 47 mm-diameter (pore size 3.0 *μ*m) polycarbonate filter (Nuclepore, Whatman). Bacterioplankton was harvested by filtration of 1 L prefiltered sea water through a filter sandwich consisting of a glass fiber filter (Whatman GF/F) and a 47 mm-diameter (pore size 0.2 *μ*m) polycarbonate filter (Nuclepore, Whatman).

 Additionally, marine phytoplankton samples were collected by employing a plankton net (pore size 55 *μ*m). The composition of the algal community was determined by microscopy of the collected samples.

### 2.2. RNA Extraction and Purification

Total RNA was extracted as described by Weinbauer et al. [15]. One 47 mm-diameter filter (pore size 0.2 *μ*m) was used per sample. Subsequently, RNA was purified employing the RNeasy Mini Kit as recommended by the manufacturer (Qiagen, Hilden, Germany).

 To remove residual DNA from RNA samples, Ambions TURBO DNase (Invitrogen, Carlsbad, USA) was used according to the instructions of the manufacturer with one modification: subsequent to a standard reaction, 0.5 *μ*L of TURBO DNase per 10 *μ*g of RNA was added to the mixture, and incubation was performed at 37°C for 15 min. Phenol/Chloroform/Isoamyl alcohol (25 : 24 : 1) was used to inactivate the DNase. 

 The presence of remaining DNA was tested by PCR using the 16S rRNA gene as a target gene for amplification. The following two primer sets were employed: 8F/518R (5′- AGAGTTTGATCCTGGCTCAG-3′ [16] and 5′-ATTACCGCGGCTGCTGG-3′ [17]) and 1055F/1378R (5′-ATGGCTGTCGTCAGCT-3′ [18] and 5′-CGGTGTGTACAAGGCCCGGGAACG-3′ [19]).

 The PCR reaction mixture (25 *μ*L) for amplification of the target gene contained 2.5 *μ*L of 10-fold Mg-free *Taq* polymerase buffer (Fermentas, St. Leon-Rot, Germany), 200 *μ*M of each of the four desoxynucleoside triphosphates, 1.75 mM MgCl_2_, 0.4 *μ*M of each primer, 1 U of *Taq* DNA polymerase (Fermentas), and approximately 100 ng of purified RNA sample as template. The following thermal cycling scheme was used: initial denaturation at 94°C for 2 min, 28 cycles of denaturation at 94°C for 1.5 min, annealing at 55°C for 1 min, followed by extension at 72°C for 40 s. The final extension was carried out at 72°C for 10 min. 

### 2.3. Synthesis of cDNA from Total RNA

cDNA was synthesized from total RNA by employing the SuperScript Double-Stranded cDNA Synthesis Kit (Invitrogen) with modifications of the first strand synthesis protocol: 10 *μ*L of total RNA (up to 5 *μ*g) were mixed with 1 *μ*L of random hexamer primers (Roche, Mannheim, Germany) and 1 *μ*L dNTP mixture containing 10 mM of each of the four desoxynucleoside triphosphates. The mixture was incubated for 10 min at 70°C and chilled on ice. Four *μ*L 5x first-strand buffer, 1 *μ*L of 0.1 M DTT, and 1 *μ*L RNA protect (Fermentas) were added, and the reaction mixture was incubated for 2 min at 25°C. Subsequently, 1 *μ*L of SuperScript II reverse transcriptase was added. The reaction was incubated for 10 min at 25°C and then for 1 h at 45°C. The generated cDNA was subjected to 16S rRNA PCR. 

### 2.4. Amplification of 16S rRNA and Pyrosequencing

To analyze archaeal diversity, the V3–V5 region of the archaeal 16S rRNA was amplified by PCR. The PCR reaction (25 *μ*L) contained 5 *μ*L of 5-fold Phusion GC buffer (Finnzymes, Vantaa, Finland), 200 *μ*M of each of the four desoxynucleoside triphosphates, 1.5 mM MgCl_2_, 4 *μ*M of each primer (Table 1), 2.5% DMSO, 1 U of Phusion High Fidelity Hot Start DNA polymerase (Finnzymes), and approximately 50 ng of cDNA. The following thermal cycling scheme was used: initial denaturation at 98°C for 5 min, 25 cycles of denaturation at 98°C for 45 s, annealing at 68°C for 45 s, followed by extension at 72°C for 30 s. The final extension was carried out at 72°C for 5 min. Negative controls were performed by using the reaction mixture without template. Primer sequences for amplification of the V3–V5 region [20] as well as 454 adaptors with the unique MIDs for each sample are listed in Table 1. The resulting PCR products were checked for appropriate size and then purified by using the peqGOLD Gel Extraction Kit (Peqlab, Erlangen, Germany) as recommended by the manufacturer. Three independent PCR reactions were performed per sample, purified by gel extraction, and pooled in equal amounts. Quantification of the PCR products was performed using the Quant-iT dsDNA BR Assay Kit and a Qubit fluorometer (Invitrogen) as recommended by the manufacturer. The Göttingen Genomics Laboratory determined the sequences of the 16S rRNA by using a Roche GS-FLX 454 pyrosequencer with Titanium chemistry (Roche, Mannheim, Germany).

### 2.5. Processing and Analysis of Pyrosequencing Derived Data Sets

Sequence data were deposited in the sequence read archive of the National Center for Biotechnology Information under accession number SRA056839. Generated 16S rRNA datasets were processed and analyzed employing the QIIME 1.4 software package and other tools [21]. The sequences were initially processed according to the denoising of 454 datasets workflow. Sequences shorter than 300 bp, with an average quality value below 25, or possessing homopolymers longer than 8 bp were removed. Afterwards, the sequences were denoised. Cutadapt was used to truncate remaining primer sequences [22]. Chimeric sequences were removed using UCHIME and the Green Genes Gold dataset as reference database [23–25]. 

 Remaining sequences were clustered employing the UCLUST algorithm [23] and the following QIIME scripts: pick_otus.py and pick_rep_set.py. The sequences were clustered in operational taxonomic units (OTUs) at 3% and 1% genetic dissimilarity. Phylogenetic composition was determined using the QIIME assign_taxonmy.py script. A BLAST alignment [26] against the most recent Silva ARB database [27] was thereby performed. Sequences were classified with respect to the taxonomy of their best hit in the ARB database. Finally, OTU tables were generated.

### 2.6. Rarefaction Analysis and Diversity Analysis

Rarefaction curves, Shannon indices [28], and Chao1 indices [29] were calculated employing QIIME scripts. In addition, the maximal number of OTUs (*n*_max⁡_) was estimated for each sample in R (version 2.15) [30] using the data derived from the QIIME rarefaction analysis and a nonlinear regression model based on Michaelis-Menten kinetics [31].

 To compare archaeal community structures across all samples based on phylogenetic or count-based distance metrics, a principal coordinate analysis (PCoA) was performed using QIIME. The following scripts were successively used to generate a phylogenetic tree at 1% genetic distance prior to PCoA calculation: align_seqs.py (PyNAST algorithm), filter_alignment.py, and make_phylogeny.py. The tree and the respective OTU table were used to generate PCoAs employing the “beta_diversity_through_plots.py” script.

## 3. Results

### 3.1. Environmental Parameters

**Figure 1:**
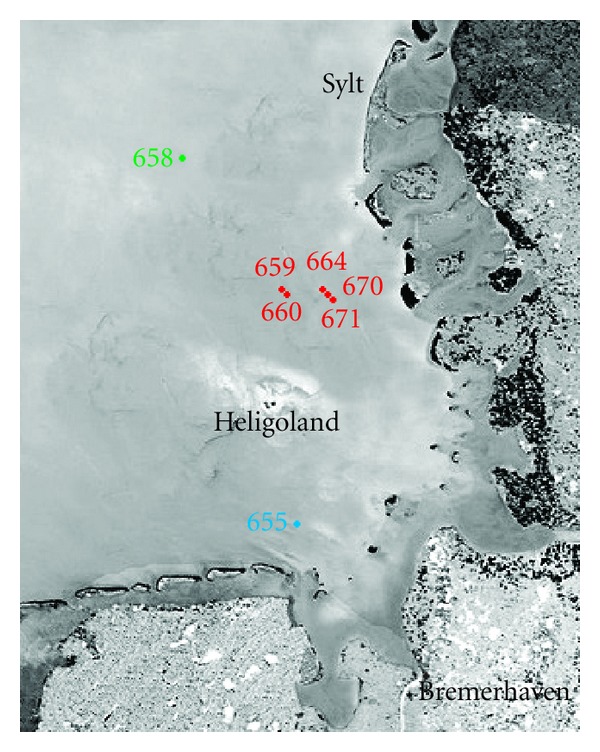
Satellite image of the German Bight showing the locations of the seven sampling sites (Image: ESA/NASA - SOHO/LASCO). Samples taken during an algal bloom (samples 659, 660, 664, 670, and 671) are shown in red. Sample 655 taken at a river outfall and sample 658 originating from outside the algal bloom in blue and green, respectively.

Marine water samples for archaeal community analysis were randomly collected at seven different locations in the German Bight (Figure 1, Table 2). Five samples (sites 659, 660, 664, 670, and 671) were taken in presence of an algal bloom. The other two samples derived from a river outfall (655) and from a site outside the algal bloom (658). The algal blooms observed during the sampling were mainly dominated by the genus *Phaeocystis*. Diatoms of the genus* Rhizosolenia* and some dinoflagellates were also identified but only in minor abundances.

 Environmental factors at all seven sampling sites were monitored employing a CTD profiler (Table 2). Temperatures and salinities ranged from 9.73 to 11.70°C and from 30.24 to 32.71 psu, respectively. The lowest temperature and highest salinity were measured at site 658. All other sites showed similar conditions. Fluorescence was higher at bloom sites due to a higher chlorophyll concentration, whereas transmission was reduced due to a higher turbidity in the water.

### 3.2. Archaeal Community Structure Revealed by 16S rRNA-Based Analysis

**Figure 2:**
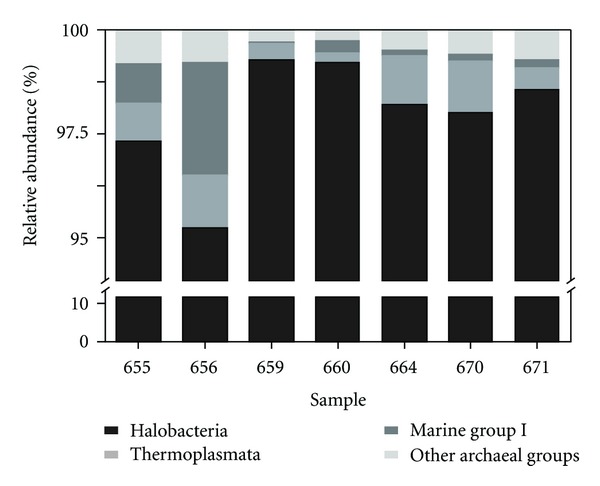
Relative sequence abundances of different archaeal phyla and classes. *Euryarchaeota*, especially *Halobacteria* (98.14%), were highly abundant. *Thermoplasmata* (0.75%) and the Marine Group 1 (0.58%) were found to some extent. All archaeal classes and groups (abundance < 0.5%) are depicted together.

To assess archaeal community structures, total RNA was extracted from the samples. Approximately 5 *μ*g of total RNA per filter were extracted from each sample. After removal of contaminating DNA and small RNAs, 0.25 to 1.5 *μ*g of RNA were used as template for cDNA synthesis. The V3–V5 region of the 16S rRNA was amplified from the generated cDNA. The resulting PCR products were subjected to pyrosequencing. Sequence processing including quality filtering, denoising, and removal of potential chimeric sequences resulted in recovery of 62,090 high quality sequences with a read length of ≥300 bp across all 7 samples. The average read length was 506 bp. The number of sequences per sample ranged from 4,301 to 23,070. We were able to assign 62,045 sequences to the domain *Archaea* and to classify all of these sequences below the domain level. The classified sequences were affiliated to three archaeal phyla with twelve archaeal classes or similar phylogenetic groups. *Euryarchaeota* was the most abundant archaeal phylum (99.25%) and *Halobacteria* the predominant class across all samples (>98.1%) (Figure 2). Most of the sequences affiliated to the *Halobacteria* (97.81%) were affiliated to uncultured members of the Deep Sea Hydrothermal Vent Group 6 (DHVEG-6) [32]. Interestingly, *Halobacteria* were more abundant in bloom samples than in other samples (Figure 2). Other archaeal groups present in all samples were the Marine Group I (*Thaumarchaeota*) [33] and the *Thermoplasamata *(*Euryarchaeota*). Sequences affiliated to the latter archaeal group belonged to the uncultured members of the CCA47 [34] group and the Marine Group II [33].

### 3.3. Diversity and Species Richness of Archaeal Communities

To determine the archaeal diversity and richness, rarefaction analyses were performed with QIIME [21]. Alpha diversity analysis was performed at the same level of surveying effort (3100 randomly selected sequences per sample). The observed OTU number in the archaeal picoplankton ranged from 252 to 454 OTUs (1% genetic distance) and from 250 to 417 OTUs (3% genetic distance) (Table 3). The maximal expectable number of clusters for every sample was determined by nonlinear regression based on the Michalis-Menten equitation. The average OTU coverages were 62.3% and 62.6% at 1% and 3% genetic distance, respectively. Shannon indices ranged from 3.74 to 7.74 (1% genetic distance) and from 3.63 to 7.62 (3% genetic distance). 

**Figure 3:**
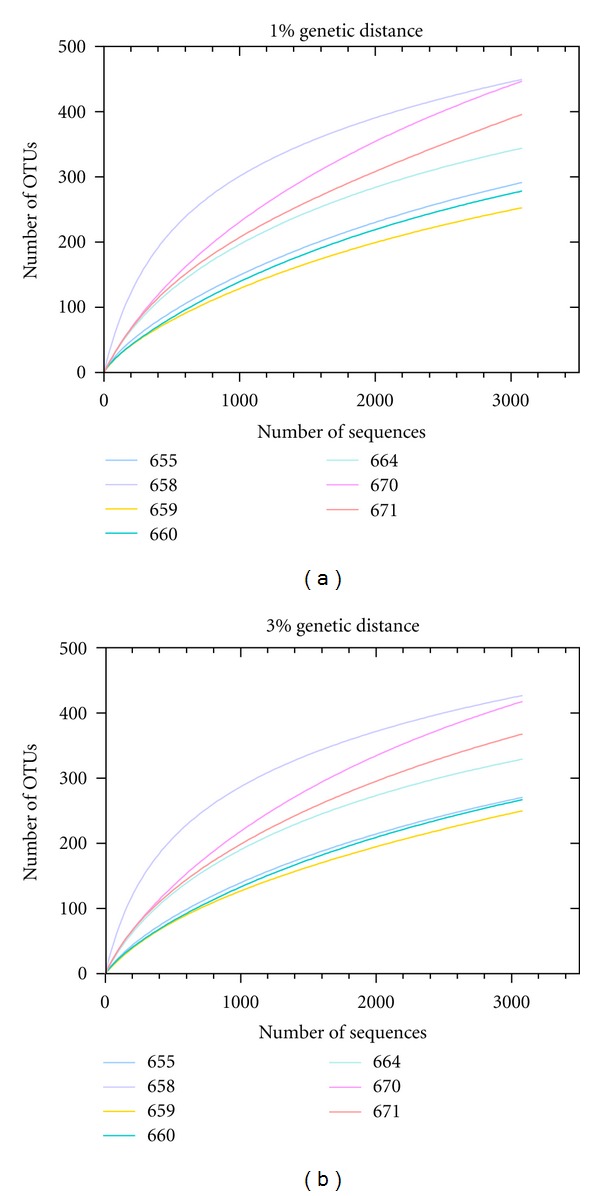
Rarefaction curves for all seven sampling sites. Curves were calculated at 1% (a) and 3% (b) genetic distance level employing QIIME [21]. Description of samplings sites is shown in Table 2.

 Comparison of the rarefaction analyses with the number of OTUs determined by Chao1 richness estimator revealed that at 1%and 3% genetic distances the rarefaction curves (Figure 3) were not saturated and the richness estimators indicated that 41.34% to 73.41% of the estimated richness, respectively, were recovered by the sequencing effort (Table 3). Thus, we did not survey the full extent of taxonomic diversity at these genetic distances, but a substantial fraction of the archaeal diversity within individual samples was assessed at genetic divergence of 3%.

### 3.4. Beta Diversity of the Bacterioplankton Community

**Figure 4:**
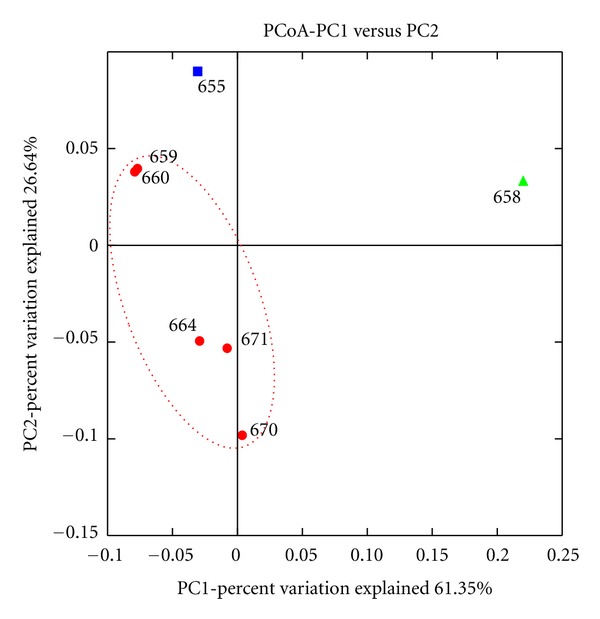
Weighted UniFrac 2D Principal Coordinate Analysis plot for beta diversity analysis. Samples taken during an algal bloom (samples 659, 660, 664, 670, and 671) are shown in red. Sample 655 taken at a river outfall and sample 658 originating from outside the algal bloom in blue and green, respectively.

Changes of the active bacterial community in response to different environmental conditions were examined by principal coordinate analysis (PCoA) (Figure 4). Surveying effort had no or little effect on diversity and community structure. However, the PCoA analysis revealed that all samples exhibiting similar environmental parameters such as temperature and salinity were assigned to one site of the plot. In addition, all bloom samples tend to cluster together. Sample 658 taken outside the algal bloom was completely separated from all other samples. 

## 4. Discussion

 Marine environments contain a high microbial biodiversity, and marine microbial communities play major roles in many biogeochemical cycles. Studies using culture-independent approaches have greatly contributed to our understanding of the extent of microbial diversity [35]. Most of these studies focused on marine bacteria, whereas very little is known on the diversity and ecology of marine *Archaea*. Recent metagenomic studies provided evidence for ammonium-oxidizing *Archaea* being capable of nitrification [36]. Some marine crenarchaeal lineages are thought to be important nitrifiers in planktonic marine systems [37]. These results indicate that *Archaea* are important players in the global nitrogen cycle. However, detailed comparative ecological studies to understand archaeal community patterns and environmental drivers that shape these communities are missing [37].

 This study focused on assessing the active archaeal community structure and richness in picoplankton samples derived from the German Bight by metatranscriptomic approaches. To our knowledge, this is the first study using an RNA-based approach combined with NGS to analyze archaeal community compositions. In addition, the obtained average read length (506 bp) is higher than in most other studies employing NGS sequencing of 16S rRNA gene amplicons [38, 39]. The majority of sequences obtained was affiliated to the *Euryarchaeota*. Sapp et al. [40] studied marine sediments derived from the Oyster Ground (North Sea) and found high abundances of members of this phylum in their samples. We identified *Halobacteria* as the most abundant archaeal group. Members of this group can grow aerobically as well as anaerobically. Large halobacterial blooms appear reddish due to production of retinal-containing rhodopsins. Rhodopsins are photoactive membrane proteins with a highly conserved tertiary structure [41] and may serve as an additional possibility to conserve energy. This is advantageous in marine environments, as the concentration of dissolved organic matter and other nutrients is usually low [42]. Most of the halobacterial sequences analyzed in this study were affiliated to the Deep Sea Hydrothermal Vent Euryarchaeotal Group 6 (DHVE-6). This group was originally described as a hydrothermal vent lineage [43]. It was later renamed Miscellaneous Euryarchaeotic Group, as members of this group were also found in marine sediment [44] and in soil [45]. Another archaeal group found in all samples was the Marine Group I (MG-I). It was originally identified by sequencing of environmental 16S rRNA genes derived from sea water [46, 47]. Members of MG-I account for large fractions of marine prokaryotic picoplankton and prokaryotic communities in deep sea water (below 3000 m). *Thermoplasmata* were the third most abundant archaeal class in the investigated samples. Most sequences were affiliated to the CCA47 group. This group was originally identified by 16S rRNA gene analysis of oxygen-depleted marine environments [48]. Later, Ferrer et al. [34] found members of this group in anoxic subsaline sediments. A few sequences assigned to Thermoplasmata were also affiliated to the Marine Group II. DeLong [33] suggested that members of Marine Group II (*Euryarchaeota*) are more abundant in temperate sea water than Marine Group I (*Crenarchaeota*) members. We found the opposite, as we recorded a higher abundance of Marine Group I members in the studied samples. Marine Group II members were almost absent in the investigated samples. One reason for this discrepancy might be that large parts of the German Bight are strongly influenced by tidal currents. Thus, these currents might whirl up archaeal cells from the sediment to the surface water, as most of the identified groups were originally described as inhabitants of marine sediments. Nonetheless, the number of studies targeting archaeal communities in the water column is substantially lower than that on marine sediments. Due to this knowledge gap, the habitat preference of these archaeal groups cannot be deduced definitely.

The impact of environmental conditions onto archaeal community composition and richness has been rarely studied. Auguet et al. [37] performed a general analytical approach to find community patterns of uncultured* Archaea* along environmental gradients or habitat types. Their results indicate that habitat types have a greater effect on archaeal community structures than other environmental conditions. All samples investigated in our study originated from almost the same habitat type, except for samples 655 and 658, which were collected at a river outfall region and outside of the algal bloom, respectively. Accordingly, all samples derived from the bloom showed an almost identical community composition. In addition, sample 655 showed a consimilar community structure. This indicates that similar environmental factors, such as temperature, salinity, and high nutrient availability during algal blooms or at river outfalls, have a similar impact onto composition of active archaeal communities.

Herfort et al. [49] studied archaeal communities in the southwestern North Sea via Denaturing Gradient Gel Electrophoresis (DGGE) and showed a positive correlation between the abundance of *Euryarchaeota *and chlorophyll concentrations, whereas the abundance of *Crenarchaeota* was negatively correlated with the chlorophyll concentration. Teeling et al. [50] investigated bacterial communities near Helgoland. They demonstrated that bacterial community structures were highly influenced by the presence of an algal bloom. In our study, we investigated the influence of algal blooms on archaeal diversity by PCoA. Sample taken in presence of a bloom shared a more similar community structure. This indicates that marine archaeal communities are also influenced by algal blooms or by environmental parameters correlated with bloom presence. We observed an increased number of *Halobacteria* in bloom samples. This might be correlated with the high amounts of organic matter in blooms. *Halobacteria* are the most active organisms with respect to organic matter degradation in hypersaline environments [37]. Thus the higher abundance of *Halobacteria* in algal bloom samples might indicate an involvement in marine organic matter degradation under high nutrient conditions found during algal blooms.

 Due to the lack of pure cultures and large comparative investigations, robust conclusions on contributions of marine archaeal communities to biogeochemical cycles cannot be drawn. In this study, we found highly diverse and active archaeal communities in the surface water of the German Bight. Their ecological role is unknown, and further research including analyses of expressed functional genes needs to be performed to unravel the role of marine *Archaea*.

## Figures and Tables

**Table 1:** Primers used for amplification of the V3–V5 region of the archaeal 16S rRNA [20].

Sample	Primer	Sequence (5′-3′)			
		454-Adaptor (Lip-A Kit)	Key	Unique MID	Archaeal 16S rRNA specific
655	ARC344F	CGTATCGCCTCCCTCGCGCCA	TCAG	ACTGTACAGT	ACGGGGYGCAGCAGGCGCGA
658	ARC344F	CGTATCGCCTCCCTCGCGCCA	TCAG	AGACTATACT	ACGGGGYGCAGCAGGCGCGA
659	ARC344F	CGTATCGCCTCCCTCGCGCCA	TCAG	AGCGTCGTCT	ACGGGGYGCAGCAGGCGCGA
660	ARC344F	CGTATCGCCTCCCTCGCGCCA	TCAG	AGTACGCTAT	ACGGGGYGCAGCAGGCGCGA
664	ARC344F	CGTATCGCCTCCCTCGCGCCA	TCAG	ATAGAGTACT	ACGGGGYGCAGCAGGCGCGA
670	ARC344F	CGTATCGCCTCCCTCGCGCCA	TCAG	CACGCTACGT	ACGGGGYGCAGCAGGCGCGA
671	ARC344F	CGTATCGCCTCCCTCGCGCCA	TCAG	CAGTAGACGT	ACGGGGYGCAGCAGGCGCGA
All	ARC915R	CTATGCGCCTTGCCAGCCCGC	TCAG	ACAGTATATA	GTGCTCCCCCGCCAATTCCT

**Table 2:** Parameters of sampling sites analyzed in this study.

Site		Latitude °N	Longitude °E	Depth (m)	*T* (°C)	Salinity (psu)	Fluorescence (mg/m^3^)	Transmission (%)
655	River outfall	53°53.729	8°02.979	2	11.09	30.24	1.21	57.2
658	No bloom	54°45.754	7°26.780	2	9.73	32.71	0.49	81.23
659	Bloom	54°27.450	7°59.360	9	10.80	30.64	2.76	60.14
660	Bloom	54°27.250	8°00.110	2	10.83	30.65	1.89	72.28
664	Bloom	54°28.400	8°11.830	2	10.90	30.76	1.14	87.28
670	Bloom	54°27.570	8°12.420	2	11.43	30.83	—*	75.72
671	Bloom	54°26.940	8°12.970	2	11.70	31.04	—*	76.59

*Fluorescence was not measured due to a malfunction of the profiler.

**Table 3:** Archaeal diversity and richness values at 1% and 3% genetic distance. Numbers of observed OTUs as well as Shannon and Chao1 values were calculated with QIIME [16]. The maximal OTU number (*n*_max_) in each sample was calculated by nonlinear modeling. Coverage was determined based on observed OTUs and *n*_max_. To compare community structures, 3100 randomly selected sequences form every sample were used.

Sample	Observed OTUs		Max. OTUs (*n*_max_)		Coverage (%)		Shannon index (H′)		Chao1	
	1%	3%	1%	3%	1%	3%	1%	3%	1%	3%
655	293	268	510	468	57.45	57.26	4.37	4.02	530	470
658	451	428	555	524	81.26	81.68	7.74	7.62	636	583
659	252	250	446	441	56.50	56.69	3.74	3.63	498	470
660	281	269	516	496	54.46	54.23	3.95	3.74	551	509
664	346	327	516	488	67.05	67.01	4.81	4.65	569	486
670	454	417	782	717	58.06	58.16	5.21	5.07	785	674
671	399	370	649	586	61.48	63.14	5.09	4.96	1227	895
